# An Audit of the First Stage of Service Development Initiatives Undertaken as Part of a Quality Improvement Process to Improve the Screening and Management of Older Adults Presenting With Delirium in Queen's Hospital (NELFT)

**DOI:** 10.1192/bjo.2024.412

**Published:** 2024-08-01

**Authors:** Jeeano Paul, Gareth Smith, Stephen O'Connor

**Affiliations:** NELFT, London, United Kingdom

## Abstract

**Aims:**

To improve the detection of delirium amongst patients aged over 65 in Queen's Hospital, and then incorporate a clearer management pathway for these patients to be treated safely with more appropriate intervention and better follow up care. As part of the management pathway, the aim was to increase the delirium referrals made to the local Dementia and Delirium Team for quicker implementation and education regarding non-pharmacological interventions in treating delirium, whilst ensuring that Psychiatric Liaison Service (PLS) referrals for delirium were also appropriate.

**Methods:**

A multi-phase approach to quality improvement and service development for patients with delirium has been adopted, and the first step is to improve the screening of patients over 65 years old with delirium and then to refer to appropriate teams accordingly. Our first intervention was changing the PLS referral form. It has been simplified with less input data required, and now includes a mandatory 4AT screening score for delirium, as well as a mandatory referral to the Dementia and Delirium Team for any patient with positive screening for delirium. The intervention was implemented in November 2023, with pre and post intervention data collected in October and December 2023 respectively. Data was collected prospectively and retrospectively using medical notes.

**Results:**

Queen's Hospital PLS received a total of 60 older adult referrals in October 2023 and 49 referrals in December 2023, of which the total proportion of referrals diagnosed with delirium was 47% and 35% respectively (12% absolute reduction). The proportion of patients referred to the PLS team with delirium, who did not require further intervention after initial assessment, had reduced by 29% (87% to 58%). The proportion of patients with delirium referred to PLS, who had also been appropriately screened and referred to the Dementia and Delirium Team prior to PLS assessment, has also increased by 4%. There has been a marked increase in total delirium referrals to the Dementia and Delirium team after intervention, from 31 referrals in October to 85 referrals in December (174% increase).

**Conclusion:**

There is an improvement in screening for delirium, with marked increase in referrals made to the Dementia and Delirium team. There is a decrease in uncomplicated delirium referrals who do not require further PLS intervention and can be appropriately managed with the Dementia and Delirium team input.

